# Force field-inspired molecular representation learning for property prediction

**DOI:** 10.1186/s13321-023-00691-2

**Published:** 2023-02-06

**Authors:** Gao-Peng Ren, Yi-Jian Yin, Ke-Jun Wu, Yuchen He

**Affiliations:** 1 Zhejiang Provincial Key Laboratory of Advanced Chemical Engineering Manufacture Technology, College of Chemical and Biological Engineering, Zhejiang University, Hangzhou, 310027 China; 2 Institute of Zhejiang University-Quzhou, Quzhou, 324000 China; 3 School of Chemical and Process Engineering, University of Leeds, Leeds, LS2 9JT UK; 4 State Key Laboratory of Industrial Control Technology, College of Control Science and Engineering, Zhejiang University, Hangzhou, 310027 China

**Keywords:** Molecular representation learning, Graph neural networks, Force field, Molecular property prediction, Protein–ligand binding affinity

## Abstract

Molecular representation learning is a crucial task to accelerate drug discovery and materials design. Graph neural networks (GNNs) have emerged as a promising approach to tackle this task. However, most of them do not fully consider the intramolecular interactions, i.e. bond stretching, angle bending, torsion, and nonbonded interactions, which are critical for determining molecular property. Recently, a growing number of 3D-aware GNNs have been proposed to cope with the issue, while these models usually need large datasets and accurate spatial information. In this work, we aim to design a GNN which is less dependent on the quantity and quality of datasets. To this end, we propose a force field-inspired neural network (FFiNet), which can include all the interactions by incorporating the functional form of the potential energy of molecules. Experiments show that FFiNet achieves state-of-the-art performance on various molecular property datasets including both small molecules and large protein–ligand complexes, even on those datasets which are relatively small and without accurate spatial information. Moreover, the visualization for FFiNet indicates that it automatically learns the relationship between property and structure, which can promote an in-depth understanding of molecular structure.

**Supplementary Information:**

The online version contains supplementary material available at 10.1186/s13321-023-00691-2.

## Introduction

Molecular representation learning is a fundamental task to learning the molecular structure–property relationship. Accurate and effective molecular representations have a pivotal role in drug discovery [1] and materials design [2] and have drawn increasing attention in the last decades [3–5]. In the early days of the study, scientists utilized hand-crafted or computed molecular descriptors, like molecular fingerprints [6] and Coulomb matrix [7], as inputs and fed them into conventional machine learning methods, like random forests [8] (RF) and support vector machine [9] (SVM). However, the manual features are not task-specific and might be easily redundant or insufficient on different tasks. This drawback can be attributed to the fact that the method based on manual features is not an end-to-end process. A more natural way to represent a molecule is to consider it as a graph, consisting of nodes and edges which are defined either via a predefined molecular graph or simply by connecting atoms that lie within a cutoff distance. Then the molecular property prediction task can be solved with the help of graph representation learning. Recent advances in graph representation learning have shown great promise in applying graph neural networks (GNNs) to model graphs. How to update node embeddings by the edges and nodes around the target node (as known as the message passing phase) is the core problem of GNNs. Kipf and Welling [10] first apply spectral graph convolutions to learning node embeddings, so-called graph convolutional network (GCN), and get the best performance on many node-level tasks. In addition to GCN, many other models use different message passing methods, such as graph attention network [11] (GAT) and graph isomorphism network [12] (GIN). These GNNs show strong competitiveness in many graph-related tasks, such as node classification and graph classification. Recent studies on molecular representation learning attempt to take advantage of these powerful GNNs.

In recent years, many effective GNNs in molecular representation learning have been developed. They make several adaptions in the inputs and network structures to make GNNs perform well on molecule datasets. Basically, these GNNs can be divided into two categories, i.e. 3D-unaware GNNs and 3D-aware GNNs. As literally, 3D-unaware GNNs do not utilize the 3D geometry of molecules, and they typically take predefined molecular graphs as inputs. One of the most famous 3D-unaware models is called DMPNN [5], which improves the GNNs’ performance on molecular property prediction tasks by incorporating bond information in the message passing phase. However, these 3D-unaware GNNs do not consider spatial information, which is very important to determine intramolecular interactions, and these interactions often have a close relationship with molecular properties. For example, the nonbonded interactions between nonbonded atom-pairs play an essential role in determining molecular functions and structures [13–15]. Although 3D-unaware models can gain the message from nonbonded atoms by stacking graph layers, they have to struggle with the over-smoothing problem. To tackle the limitations of 3D-unaware GNNs, a series of 3D-aware GNNs have been developed. These 3D-aware GNNs are dedicated to utilizing accurate 3D information to obtain more powerful molecular representations. To include both bonded and nonbonded interactions, these models construct 3D graphs without predefined edges to capture all the interactions within the cutoff distance. The first obstacle that 3D-aware GNNs encounter is how to maintain translational and rotational invariance of molecular spatial information. There have been many attempts for solving this problem. The DTNN [16] and SchNet [17] maintain these invariances by computing pairwise distance; the DimeNet [18] adds considerations of angles between bond pairs by using a directional message passing scheme; the SphereNet [19] unifies SchNet and DimeNet and establishes a spherical message passing scheme using distance, angle, and dihedral. As for expansions of spatial information, the SchNet and DTNN use radial basis functions (RBF) to project the distance or angle into Gaussian space. DimeNet [18] and SphereNet [19] project the spatial information in the solution space of the Schrödinger equation, i.e., learnable RBF and spherical Bessel functions (SBF). These 3D-aware GNNs have shown their excellence in quantum property prediction; however, these models sometimes show worse performance than 3D-unaware GNNs, especially on datasets that are relatively small and without accurate spatial information [20, 21]. This shortage can be attributed to two reasons. First, these models ignored predefined bonded information, which makes these models need a great number of data points to learn the atom-pair relationship, such as whether two atoms are bonded or nonbonded. Second, these models often expand spatial information on a sophisticated space, which makes them sensitive to the accuracy of 3D information. When they take datasets that do not have accurate information, they will magnify this inaccuracy and generate biased molecular representations. However, the fact is that most labeled molecular datasets are relatively small and lack accurate spatial information since it is very time-consuming to get labeled data points and the best conformers. Therefore, it is worth exploring a 3D-aware method that can utilize the most prior knowledge of molecules, while with less sensitivity to the accuracy of the 3D information, to obtain powerful molecular representations.

In this paper, we construct a force field-inspired neural network (FFiNet) that can utilize all the interactions in molecules. Force field, which is a simple approximation to calculate the potential energy in molecules, divides all the interactions in molecules into four parts, i.e., bonded interactions, angle bending interactions, torsion interactions, and nonbonded interactions. Intuitively, these interactions can denote the importance of source atoms toward target atoms, which can be associated with graph attention mechanisms. Following this idea, we construct a novel attention-based message passing scheme that calculates the importance scores of source atoms within three hops according to their interactions with target atoms. The proposed model shows state-of-the-art performance on extensive molecular property benchmark tasks, even on those small datasets without accurate information. Moreover, we also applied our model to represent large protein–ligand complexes and predict their property (i.e. binding affinity). The result shows that FFiNet can outperform all the baselines on the PDBBind dataset, which further indicates the competence of FFiNet for molecular representation learning. Furthermore, the visualization of the learned node features and attention weights agrees well with the intuition of the relationship between chemical molecular structure and property.

## Results

### Network architecture

**Fig. 1:**
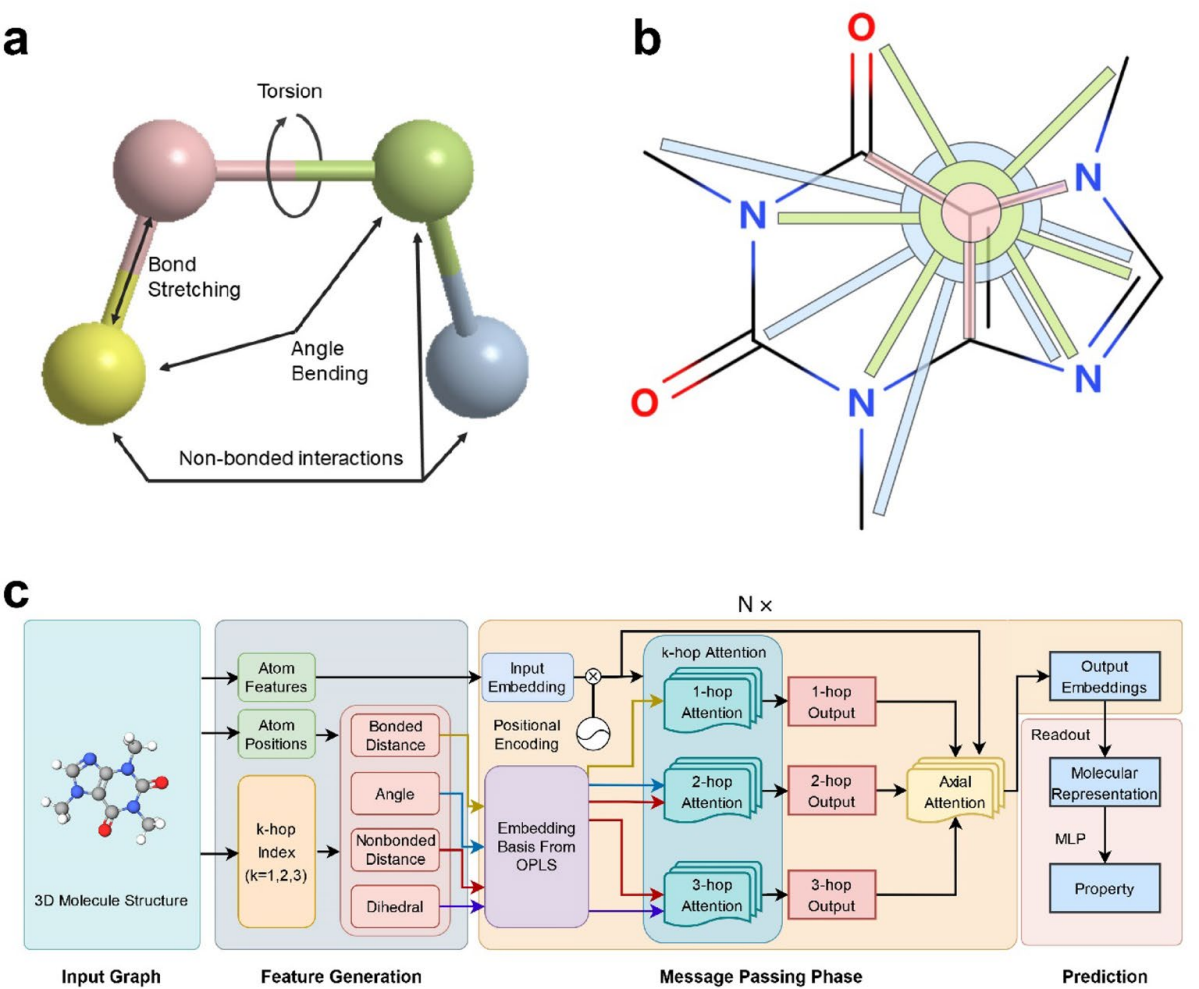
Illustration of the proposed FFiNet model. **a** Illustration of four types of potential energy terms in a molecule. **b** Illustration of message passing in FFiNet. Message from 1-hop nodes (pink), 2-hop nodes (green), and 3-hop nodes (blue) are used to inform the update to the embedding of the carbon atom located at the junction of two rings. **c** The model structure of FFiNet. The model takes 3D molecular structure as input and generates atom features and spatial information. Then the model takes the embedded atom features and spatial information to the k-hop attention module and gets k-hop outputs. Positional encoding and axial attention are applied to get the final node embeddings to distinguish the nodes from different hops. Finally, the model uses a readout function and a multi-layer perceptron (MLP) to predict molecular properties

The force field divides the total potential energy into four terms [22]:

1 $$E={E}_{\mathrm{bond}}+{E}_{\mathrm{angle}}+{E}_{\mathrm{torsion}}+{E}_{\mathrm{nonbonded}}$$

where ${E}_{\mathrm{bond}}$ is the bond stretching energy, ${E}_{\mathrm{angle}}$ is the angle bending energy, ${E}_{\mathrm{torsion}}$ is the torsional energy, and ${E}_{\mathrm{nonbonded}}$ is the nonbonded interaction energy. Figure 1a shows a more intuitive explanation. As shown, there is bond stretching between bonded atoms, angle bending between bonds, torsion angle between two atomic pairs (i.e., the dihedral angle between two planes defined by pairs of bonds), and nonbonded interactions between nonbonded atoms. As previously introduced, the main purpose of GNNs is to update node embeddings. Therefore, Fig. 1a can also be interpreted differently: if the yellow node is the node to update embedding, it involves the bond stretching with the pink node (1-hop node), the angle bending and nonbonded interactions with the green node (2-hop node), and the torsion and nonbonded interactions with blue node (3-hop node). From this intuition, we build up the FFiNet model aggregating node information and corresponding spatial information from nodes within 3-hop instead of only 1-hop to update node embeddings (Fig. 1b). Setting the receptive filed of GNNs as three hops can not only help include nonbonded information, angle, and dihedral information but also reduce computational cost compared to global molecular representation learning models (such as Graphormer [23]). More advantages for setting receptive field as three hops can be seen in the Ablation studies section and Additional file 1: Fig. S2. As shown in Fig. 1b, the message passing phase is involved within three hops nodes (i.e. 1-hop nodes (pink), the 2-hop nodes (green), and the 3-hop nodes (blue)). The contributions of these nodes to the target nodes are measured by two types of attention mechanisms, i.e., k-hop attention and axial attention. The k-hop attention is to calculate the importance scores with target nodes of source nodes in the same hop by incorporating the functional form of intramolecular potential energy, and the axial attention is to calculate the scores of source nodes in the different hops.

The overall architecture can be seen in Fig. 1c. First, we extract the k-hop index of molecules, including the 1-hop index of pair nodes (also called edge index), 2-hop index of three atoms chain, and 3-hop index of four atoms chain. Then the distance, angle, and dihedral can be calculated with the atomic positions and the indexes. To include the spatial information in the FFiNet model, we refer to the functional form in the force field. Since most force fields have a very similar functional form [24–26], we choose the widely used OPLS force field [27] to help embed the spatial information in the model. The formulas of four interaction terms in the OPLS force field are listed below.

As shown in Table 1, the embedding basis can be extracted from the functional form in the OPLS force field. Then, we include the spatial information in the k-hop attention mechanism (similar to GATv2 [28], the novel variants of GAT) to distinguish the contributions of the same hop nodes. The attention mechanism can be written as:

2 $${e}_{ij}^{k}={{\varvec{a}}}_{k}^{\mathrm{T}}\mathrm{LeakyReLU}\left(\left({\sum }_{r\in {I}_{k}\left(i,j\right)}{{\varvec{W}}}_{r}{{\varvec{h}}}_{r}\right)\odot \left({{\varvec{W}}}_{k}{{\varvec{m}}}_{k}\right)\right)$$

where ${e}_{ij}^{k}$ denotes the attention score between the k-hop atom $j$ and the target atom $i$, ${I}_{k}\left(i,j\right)$ denotes the k-hop index with $i$ as the target index and $j$ as the source index, *r* denotes the node index in the k-hop index, ${{\varvec{h}}}_{r}$ denotes the input node representations, ${{{\varvec{a}}}_{k},{\varvec{W}}}_{r},{{\varvec{W}}}_{k}$ are learnable parameters, and ${{\varvec{m}}}_{k}$ denotes the embedding basis of different hops, i.e. bonded embedding basis for one-hop nodes, the concatenation of angle and nonbonded embedding basis for two-hop nodes, and the concatenation of torsion and nonbonded embedding basis for three-hop nodes. After the k-hop attention scores are calculated, a softmax function is used to normalize scores across the same hop nodes. Since the above attention mechanism contains no information about the relative position of different hop nodes, we introduce the positional encoding module to the input node embeddings. We use sine and cosine functions of different frequencies as position encodings just like the transformer [29] did, and multiply them to the input embeddings. After calculating the normalized attention scores, the new node embeddings are generated by a weighted average of the node features of the same hop neighbors. Finally, three new node embeddings (k-hop outputs) are generated, called 1-hop output, 2-hop output, and 3-hop output, respectively.

**Table 1:** The OPLS force field potential energy terms and corresponding embedding basis

OPLS potential energy terms	Embedding basis
${E}_{\mathrm{bond}}={\sum }_{\mathrm{bonds}}{K}_{r}{\left(r-{r}_{\mathrm{eq}}\right)}^{2}$	$\{r, {r}^{2}\}$
${E}_{\mathrm{angle}}={\sum }_{\mathrm{angles}}{K}_{\theta }{\left(\theta -{\theta }_{\mathrm{eq}}\right)}^{2}$	$\{\theta , {\theta }^{2}\}$
${E}_{\mathrm{torsion}}={\sum }_{\mathrm{dihedrals}}\frac{{V}_{\phi ,1}}{2}\left[1+\mathrm{cos}\left(\phi +{f}_{\phi ,1}\right)\right]+\frac{{V}_{\phi ,2}}{2}\left[1-\mathrm{cos}\left(2\phi +{f}_{\phi ,2}\right)\right]+\frac{{V}_{\phi ,3}}{2}\left[1+\mathrm{cos}\left(3\phi +{f}_{\phi ,3}\right)\right]$	$\{\mathrm{cos}\phi ,\mathrm{ cos}2\phi ,\mathrm{cos}3\phi ,\mathrm{sin}\phi ,\mathrm{sin}2\phi ,\mathrm{sin}3\phi \}$
${E}_{\mathrm{nonbonded}}={\sum }_{i}{\sum }_{j}\left[\frac{{q}_{i}{q}_{j}}{{r}_{ij}}+4{\epsilon }_{ij}\left(\frac{{\sigma }_{ij}^{12}}{{r}_{ij}^{12}}-\frac{{\sigma }_{ij}^{6}}{{r}_{ij}^{6}}\right)\right]{f}_{ij}$	$\{{r}^{-1},{r}^{-12}, {r}^{-6}\}$

$r$ is bond length, $\theta$ is valence angle, and $\phi$ is the dihedral angle; other variables are parameters of the force field (specific definitions can be found in Reference [27])

From the intuition that different hop atoms have different forces with the destination atom, we use axial attention to determine the contributions of these three outputs. We adopt dot production of k-hop outputs and input embeddings to get the axial attention score of different hops. Then the node embeddings are updated by a weighted sum of these three outputs. After the node updating steps are iterated for N steps, a readout function is applied to aggregate the output embeddings of all nodes into a graph-level representation. More precisely, we apply weighted sum and max-pooling to the node representations and concatenate the results as the graph representation. Finally, we use an MLP to give a final molecular property prediction. Besides, the model uses some performance-improving mechanics, such as residual connect [30], dropout [31], and layer normalization [32]. More detailed information about the FFiNet model can be referred to Additional file 1: Note 1.

### Performance of methods on the property prediction of small molecules

We experiment on ten molecular property benchmarks from MoleculeNet [33], including small datasets without spatial information (ESOL, Lipophilicity, FreeSolv, BACE, BBBP, ClinTox, Tox21, SIDER, HIV) and large datasets with spatial information (QM9). Below, we include a short description of these tasks.

- ESOL, FreeSolv, Lipophilicity: Regression tasks for predicting log water solubility (ESOL), hydration free energy of small molecules in water (FreeSolv), and octanol/water distribution coefficient (Lipophilicity). The datasets have 1128, 642, and 4200 molecules, respectively. These datasets represent the molecules in SMILES format, which does not include the spatial information of molecules.
- BACE, BBBP, HIV: Binary classification tasks for predicting inhibition of human β-secretase 1 (BACE), the ability to penetrate the blood–brain (BBBP) inhibition of HIV replication (HIV). The datasets have 1513, 2039, and 41,127 molecules, respectively. These datasets represent the molecules in SMILES format.
- QM9: Quantum mechanical properties regression tasks with 12 targets. The dataset has 133,885 molecules and all the molecules in the dataset have accurate spatial information.
- ClinTox, Tox21, SIDER: Multi-class classification tasks for predicting toxicity (ClinTox, Tox21) and sider effects of drugs (SIDER). The datasets have 1478, 7831, and 1427 molecules, respectively. The datasets have 2, 12, and 27 tasks, respectively. These datasets represent the molecules in SMILES format.

Many models can be used to give predictions for these benchmarks. Basically, these models can be divided into descriptors-based methods and graph-based methods. In this work, we compare FFiNet with multiple methods, including (1) SVM and RF, which are traditional machine learning (ML) tools for classification and regression; (2) GIN [12], GCN [10], which are commonly used GNNs for graph representation learning; (3) GATv2 [28], which is a novel graph attention network with more expressive dynamic attention than original GAT; (4) DMPNN [5], which is a well-designed GNN for molecular property prediction. (5) DimeNet [18], which is a recent excellent method for modeling on 3D molecule graphs. In order to give a comparison with these baseline models, root mean square error (RMSE) and area under the receiver operating characteristic curve (ROC-AUC) are applied to evaluate the performance of the models on the regression tasks (except QM9) and classification tasks, respectively. Following the most recent works [17, 34, 35], we report the mean absolute error (MAE) on QM9 dataset. The results of the proposed FFiNet model and various baseline models on the selected benchmark datasets are listed in Tables 2 and 3. It can be seen that the graph-based methods perform better than the ML-based methods on most benchmarks. Unlike the ML-based methods can only operate on given molecule features, the graph-based methods can generate task-specific molecule features by aggregating atom features.

**Table 2:** Performance comparison on property prediction of small molecules (regression tasks)

Metric	RMSE ↓			MAE ↓
Dataset	ESOL	Lipophilicity	FreeSolv	QM9
SVM	1.128 (0.081)	0.785 (0.032)	2.283 (0.324)	**–**^a^
RF	1.206 (0.034)	0.859 (0.030)	2.093 (0.566)	14.584 (0.047)
GATv2	0.578 (0.031)	0.618 (0.014)	1.017 (0.122)	3.449 (0.146)
GIN	0.619 (0.044)	0.756 (0.007)	1.136 (0.235)	4.972 (0.263)
GCN	0.778 (0.101)	0.899 (0.035)	1.582 (0.325)	10.158 (0.236)
DMPNN	0.665 (0.052)	0.596 (0.050)	1.167 (0.150)	3.101 (0.010)
DimeNet	0.730 (0.154)	0.699 (0.096)	0.890 (0.191)	**0.748 (0.065)**
FFiNet	**0.551 (0.030)**	**0.579 (0.022)**	**0.756 (0.138)**	1.803 (0.102)

The SOTA results are shown in bold. Standard deviations are in brackets

^a^As SVM on QM9 is too time-consuming, we could not finish on time

**Table 3:** Performance comparison on property prediction of small molecules (classification tasks)

Metric	ROC-AUC ↑					
Dataset	BACE	BBBP	HIV	Tox21	SIDER	ClinTox
SVM	0.811 (0.054)	0.829 (0.060)	0.627 (0.009)	0.822 (0.006)	0.682 (0.013)	0.669 (0.092)
RF	0.815 (0.049)	0.790 (0.062)	0.645 (0.015)	0.769 (0.015)	**0.684 (0.009)**	0.713 (0.056)
GATv2	0.843 (0.035)	0.893 (0.021)	0.818 (0.012)	0.840 (0.026)	0.618 (0.036)	0.694 (0.146)
GIN	0.850 (0.031)	0.890 (0.007)	0.786 (0.031)	0.824 (0.015)	0.619 (0.009)	0.753 (0.132)
GCN	0.829 (0.037)	0.895 (0.003)	0.752 (0.020)	0.788 (0.025)	0.624 (0.019)	0.615 (0.013)
DMPNN	0.878 (0.032)	0.913 (0.026)	0.816 (0.023)	0.845 (0.015)	0.646 (0.016)	0.894 (0.027)
DimeNet	0.832 (0.023)	0.822 (0.040)	0.724 (0.016)	0.758 (0.019)	0.626 (0.008)	0.738 (0.020)
FFiNet	**0.891 (0.016)**	**0.916 (0.012)**	**0.828 (0.010)**	**0.852 (0.009)**	0.656 (0.017)	**0.919 (0.021)**

The SOTA results are shown in bold. Standard deviations are in brackets

Tables 2 and 3 shows that the proposed FFiNet model performs better than the GATv2, GIN, GCN, and DMPNN models on all the datasets. A possible explanation might be that the latter models do not consider spatial information and nonbonded interactions. Although the traditional GNNs can get the message from nonbonded atoms by stacking layers, they usually only aggregate the message from no more than five hops atoms due to the problem of over-smoothing [36–38]. Besides, their message passing scheme only propagates messages along with chemical bonds, which is inconsistent with the intuition that atoms can interact with others without chemical bonds. Compared with 3D-unaware GNNs, the FFiNet can leverage spatial information and include nonbonded interactions explicitly. As for 3D-aware GNNs, although DimeNet outperforms other models on the QM9 dataset which is large and has accurate spatial information, it does not perform better on small datasets without accurate spatial information than the FFiNet, even compared with the traditional 3D-unaware GNNs. The reason why DimeNet performs better than FFiNet on the QM9 dataset can be attributed to the simple expansion of spatial information in FFiNet, which, however, also means our model is less sensitive on accurate 3D molecular geometry. Moreover, since DimeNet loses the predefined bonded information, they need a number of data points to learn this information, leading to bad performance on small datasets.

### Performance of methods on the property prediction of protein–ligand complexes

**Fig. 2:**
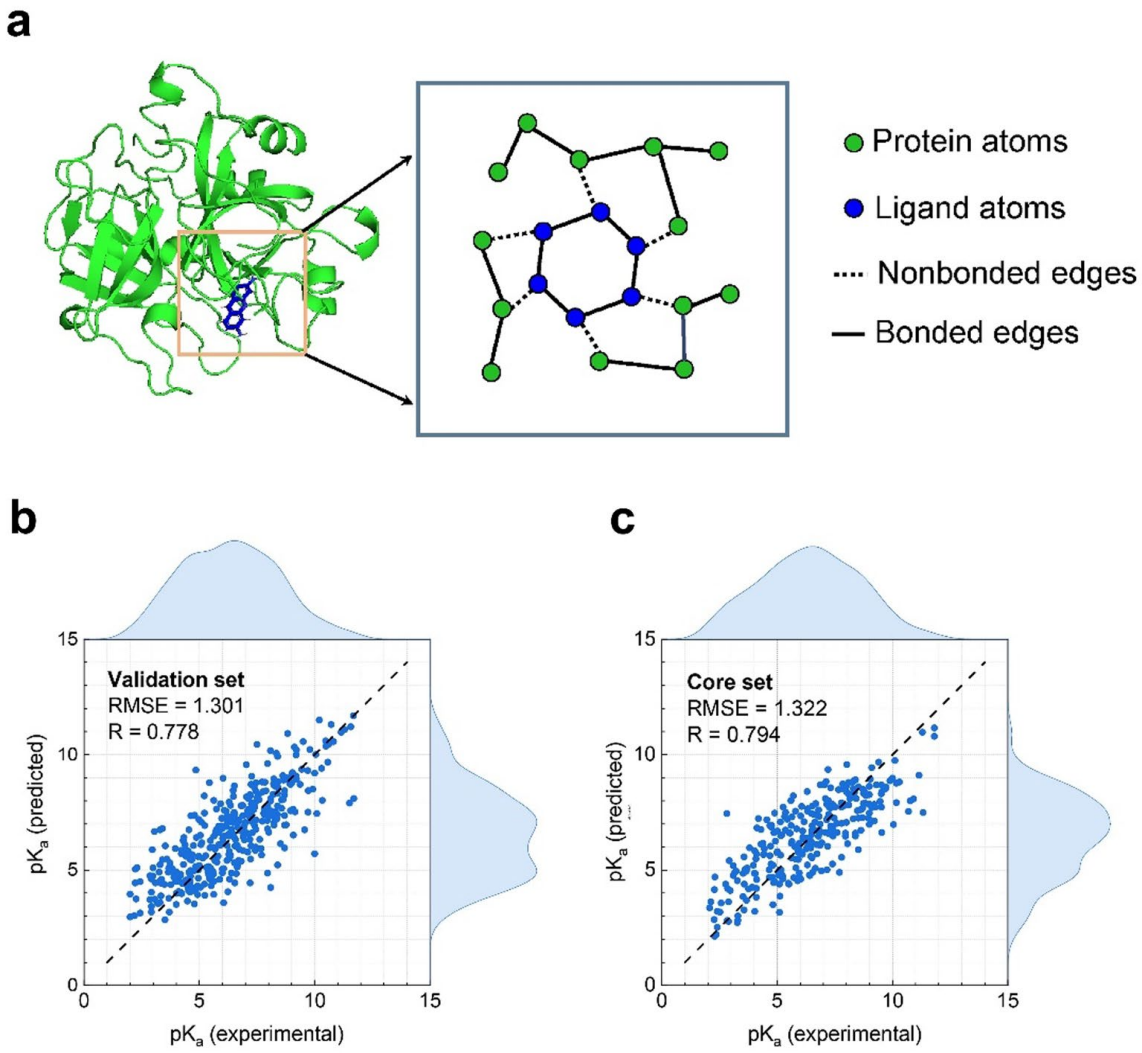
FFiNet on PDBBind v2016. **a** FFiNet constructs a complex graph by adding nonbonded edges if the atoms in protein and the atoms in ligand are close enough. **b** FFiNet-predicted pK_a_ against the experimentally measured pK_a_ on the validation set. **c** FFiNet-predicted pK_a_ against the experimentally measured pK_a_ on the core (test) set

Apart from molecular property prediction of small molecules, the prediction of protein–ligand binding affinity is also of vital importance in computational drug discovery [39]. The first step of predicting protein–ligand properties is to get protein–ligand complex representations. In the early days of molecular representation study, scientists first generate protein–ligand descriptors including atom features or intermolecular features [40, 41], then they introduce these features into traditional machine learning methods (such as RF and SVM) or modern deep learning methods (such as OnionNet [41]) to give the final prediction. To achieve end-to-end predictions, Pafnucy [42] treats a protein–ligand complex as a 3D grid and uses a 3DCNN to predict its binding affinity. With the development of GNNs, GraphDTA [43] represents a protein–ligand complex as the combination of a ligand graph and a protein sequence, and applies GNNs and convolutional neural networks (CNNs), respectively. With the development of 3D-aware GNNs, SIGN [44] constructs complex interaction graphs based on atom positions and cutoff distance. The model, including polar-inspired graph attention layers and pairwise interactive pooling, can achieve SOTA performance on affinity predictions. To demonstrate the extensibility of our model, we construct complex graphs which do not lose bonded information (as shown in Fig. 2a). We merge predefined protein graphs and predefined ligand graphs by generating nonbonded edges if their atom-pair distance is less than the cutoff threshold of 5 Å. This cutoff distance has been proved an appropriate distance to achieve a trade-off between better molecular representations and less computational cost [44]. In order not to change the basic structure of FFiNet, we concatenate nonbonded edges and bonded edges as total edge index and distinguish them by edge attribute indicators. As for the attention scores of nonbonded edges, we use the nonbonded basis for their nonbonded distances.

Herein, we use a well-known protein–ligand binding affinity benchmark (PDBBind) [45] to evaluate the performance of our model and baselines. PDBBind benchmark is a regression task for predicting binding affinities for bio-molecular complexes. The dataset provides detailed 3D Cartesian coordinates of atoms in both ligands and their target proteins. In our experiment, we choose different examples of the refined set (4057 complexes) and core set (290 complexes) to train the FFiNet model and the core set with the best quality as the test set. We use four evaluation metrics in our experiments, i.e. RMSE, MAE, Pearson correlation coefficient (R), and the standard deviation (SD) introduced in Pafnucy [42]. The performance of all baselines is from Li et al. [44].

As shown in Fig. 2b and c, the predicted and experimental values are highly correlated and their distributions are consistent on the validation and test sets, highlighting the impressive predictive capacity of the FFiNet model. To be more expressive, we list performance comparisons of various models in Table 4. As shown in Table 4, FFiNet can achieve SOTA performance on the PDBBind dataset. This is because our model is able to retain prior knowledge of the complexes to the greatest extent possible, such as spatial information, bonded information, and nonbonded information. Moreover, the protein–ligand binding affinity is dominated by nonbonded interactions such as Van der Waals forces and hydrogen bonds [46]. Therefore, the FFiNet with the explicit consideration of nonbonded interactions can perform well on the PDBBind dataset, which also indicates that the FFiNet could be easily transferred to predict other biomacromolecules or protein–protein complexes properties.

**Table 4:** Performance comparison on PDBBind 2016

Method		PDBBind 2016			
		RMSE ↓	MAE ↓	SD ↓	R ↑
ML-based methods	RF	1.446 (0.008)	1.161 (0.007)	1.335 (0.010)	0.789 (0.003)
	SVR	1.555 (0.000)	1.264 (0.000)	1.493 (0.000)	0.727 (0.000)
CNN-based methods	OnionNet	1.407 (0.034)	1.078 (0.028)	1.391 (0.038)	0.768 (0.014)
	Pafnucy	1.585 (0.013)	1.284 (0.021)	1.563 (0.022)	0.695 (0.011)
GraphDTA methods	GCN	1.735 (0.034)	1.343 (0.037)	1.719 (0.027)	0.613 (0.016)
	GAT	1.765 (0.026)	1.354 (0.033)	1.740 (0.027)	0.601 (0.016)
	GIN	1.640 (0.044)	1.261 (0.044)	1.621 (0.036)	0.667 (0.018)
	GAT-GCN	1.562 (0.022)	1.191 (0.016)	1.558 (0.018)	0.697 (0.008)
GNN-based methods	DMPNN	1.493 (0.016)	1.188 (0.009)	1.489 (0.014)	0.729 (0.006)
	DimeNet	1.453 (0.027)	1.138 (0.026)	1.434 (0.023)	0.752 (0.010)
	SIGN	1.316 (0.031)	**1.027 (0.025)**	1.312 (0.035)	0.797 (0.012)
Ours	FFiNet	**1.310 (0.012)**	1.056 (0.006)	**1.304 (0.014)**	**0.801 (0.005)**

The SOTA results are shown in bold. Standard deviations are in brackets

### Ablation studies

Since our model makes several changes compared with the previous GNNs, we do some ablation experiments to verify the necessity of each added module (Table 5). To study the effect of the proposed k-hop attention mechanism, we remove the 3-hop attention (the model called FFiNet-2hop) or remove both the 2-hop attention and the 3-hop attention (the model called FFiNet-1hop), then test their performance on all the datasets in this work. As shown, the model performances increase in the order from FFiNet-1hop, FFiNet-2hop to FFiNet on most datasets. The result proves that the 2-hop and 3-hop attention can help increase the model performance. To study the effect of the proposed axial attention mechanism, we remove the axial attention in the model; instead, we simply sum k-hop outputs to get the final node embedding (the modified model called FFiNet-no-axial). Table 5 shows that the FFiNet-no-axial model performs worse than FFiNet on all datasets. The result can be attributed to that simply summation does not consider the different contributions from nodes of different hops. However, with axial attention, the model can distinguish different hops contributions by assigning different scores to k-hop outputs.

**Table 5:** Ablation studies

Method	Regression tasks (RMSE) (RMSE, lower is better)				Classification tasks (ROC-AUC, higher is better)	
	ESOL	Lipophilicity	FreeSolv	PDBBind	BACE	BBBP
FFiNet-no-axial	0.638 (0.048)	0.603 (0.037)	1.019 (0.283)	1.624 (0.045)	0.869(0.010)	0.847 (0.021)
FFiNet-1hop	0.614 (0.047)	0.685 (0.088)	0.951 (0.010)	1.437 (0.031)	0.876 (0.024)	0.897 (0.015)
FFiNet-2hop	0.607 (0.039)	0.648 (0.076)	0.808 (0.148)	1.392 (0.044)	0.856 (0.022)	0.907 (0.019)
FFiNet	**0.551 (0.030)**	**0.579 (0.022)**	**0.756 (0.138)**	**1.310 (0.012)**	**0.891 (0.016)**	**0.916 (0.012)**

The SOTA results are shown in bold. Standard deviations are in brackets

### Model interpretation

**Fig. 3:**
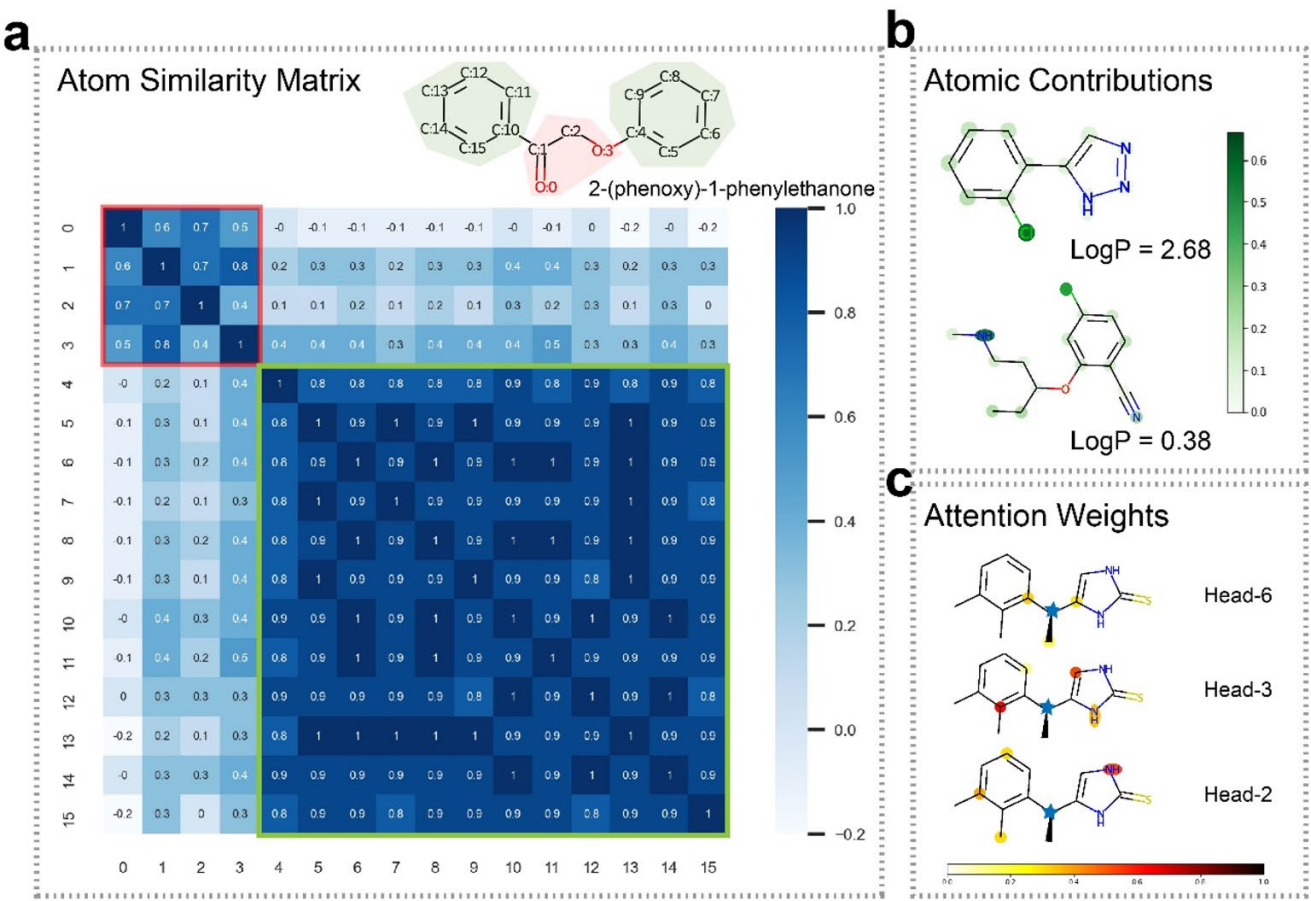
Visualization of FFiNet. **a** Heat maps of the atom similarity matrix for the compound 2-(phenoxy)-1-phenylethanone. The atom similarity is obtained by calculating the Pearson correlation coefficient for the output state vectors of the final layer. The atoms in the molecule are automatically separated into two clusters after training. The part shown in the red square corresponds to the area highlighted in red in the molecule, and the part shown in the green square corresponds to the areas highlighted in green in the molecule. **b** The atomic contributions for the lipophilicity. The chlorine atom in the first molecule and the secondary amine atom in the second molecule get the most weight. The LogP of the molecule above and the molecule below are 2.68 and 0.38, respectively. **c** The attention weights of the atoms within 3-hop of the target atom. The target atom is denoted by a blue pentagram. Different attention heads can perceive the message of atoms at different hops

The FFiNet has achieved state-of-the-art performance on a variety of molecular prediction tasks. Therefore, it is worth investigating the interpretability of the model. Model interpretation can help us find how the model learns the molecule representations and what happened during the model learning process. Although neural networks are often called “black boxes”, we can explore the interpretability of the model by visualizing the features and attention weights. Taking the lipophilicity dataset as an example, we choose some molecules to visualize atom similarity, atom contributions, and attention weights (Fig. 3). Since the GNN models learn mainly by updating the node embeddings, the final node embeddings are essential for predicting molecular property. To explore whether the model has learned some pattern after the message passing phase, we plot the heat map of the atom similarity matrix by calculating the Pearson correlation coefficient between atom pairs (Fig. 3a). Taking the molecular structure of 2-(phenoxy)-1-phenylethanone as an example, we can find that there are two parts with high values in the matrix. The part shown in the red square corresponds to the area highlighted in red in the molecule, and the part shown in the green square corresponds to the areas highlighted in green in the molecule. This pattern clearly suggests that the model can distinguish the benzene rings and the carbon–oxygen chain after training. In addition to this molecule, more examples of atom similarity heat maps are shown in Additional file 1: Figs. S3–5, which provide further evidence that the FFiNet can distinguish functional groups in molecules.

Figuring out which parts of a molecule play a more important role in a certain property is helpful for the chemist to design molecules with the desired property and learn the nature of the property. Since our model uses weighted sum pooling and max pooling as readout functions, the weight that each atom obtained can represent the importance of atoms for a certain property to some degree. Taking the lipophilicity property as an example, we plot a heat map over molecules showing the atom contributions to the property (Fig. 3b). As shown, for the molecule above, the chlorine atom shows the largest atomic contribution; for the molecule below, the chlorine atom and the secondary amine atom shows large atomic contribution values. These findings are consistent with that of Wildman et al. [47] who use the atomic contributions method to give a prediction of lipophilicity. Their results show that the chlorine atom has a large positive value of contribution for lipophilicity and the secondary amine atom has a large negative value of the contribution. And as Fig. 3b shows, the partition coefficient (LogP) which describes the lipophilicity of molecule of the molecule above and the molecule below are 2.68 and 0.38, respectively. This consistency indicates that the FFiNet can correctly assess the importance of atoms to the molecular property. Additional examples can be found in Additional file 1: Fig. S6. To further explore how the attention mechanisms in FFiNet work, we multiply the axial attention weights and k-hop attention weights between the target atom and the query atoms as total attention weights, then plot the attention weights between the target atom (denoted by a blue pentagram) and the atoms within 3-hop on different heads (Fig. 3c). As shown, Head-6, Head-3, and Head-2 give higher weights to 1-hop atoms, 2-hop atoms, and 3-hop atoms, respectively. The result indicates that the target atom gets comprehensive information about the surrounding atoms by assigning different attention weights to different heads in FFiNet.

## Conclusion

Molecular property is of vital importance in various fields, e.g., pharmaceutical and chemical engineering. There have been many attempts at the accurate prediction of molecular properties. Early attempts use molecule descriptors as input features and feed them into RF, SVM, or neural networks (NN). However, the generation of descriptors is time-consuming, and the descriptors-based methods cannot obtain satisfactory results due to the unchanged molecule representation. Recently, more and more research has been on applying machine learning to graphs, and many graph-based network architectures have been constructed, e.g., GCN, GAT, GIN. However, these models are designed mainly for citation graphs and knowledge graphs, which are not mainly designed for molecular representation learning. To this end, a series of well-designed GNNs are proposed for molecular properties, e.g., DMPNN, DimeNet. However, these models also do not uncover all the information about a molecule. Some of them lose spatial information, and some of them lose bonded or nonbonded information.

In this work, we construct a theory-guided network based on the force field. Inspired by the force field, we devise an attention network including all the interactions in a molecule, i.e., bonded and nonbonded interactions. The bonded interactions include bond stretching, angle bending, and torsion, corresponding to spatial information of distance, angle, and dihedral. We utilize the spatial information with guidance from the functional form in the force field to include all these intramolecular interactions. Since FFiNet is less sensitive to the accuracy of spatial information, it enables quick screening for those molecules without accurate spatial information. In contrast to other tested models, FFiNet performs well across a wide range of molecular property prediction tasks. Even in the task with large molecule graphs, the model can also perform well. Moreover, the visualization of the hidden state, atom contributions, and attention weights provides access to model interpretation. The results indicate that our theory-based model has the interpretability to relate property with molecular structure.

Our FFiNet can easily extend to other complex tasks, such as protein–protein interactions prediction or DNA-binding proteins prediction. These tasks need a model that has a sizeable receptive field and can model intramolecular interactions since their molecular size is very large and the nonbonded interactions often play a leading role in the properties. One potential limitation of the model is that in our message passing phase, a node involves two attention mechanisms, which need more parameters. Therefore, our following work is to find a generic global attention mechanism which can distinguish different hop nodes and nodes in the same hop with spatial information. All in all, the design philosophy of our model has great potential for driving the development of molecular representation learning.

In addition, FFiNet has great potential to cover other more specific tasks by changing the applied force field. By extracting the embedding basis from other functional forms of force fields, it is simple to utilize other force fields for better representations of a certain class of molecules. For example, when the task is about macromolecules (such as the property prediction task of proteins), the functional form of CHARMM [48] and OPLS-AA [49] could be utilized to embed spatial information. When studying the property of the condensed phase, the functional form of GROMOS [50] can provide a potential embedding basis for better predictions. Meanwhile, in this work, the function of force fields in FFiNet is mainly to provide a series of embedding basis for spatial information. Future work could consider using the force field parameters to help obtain more powerful molecular representations with more prior knowledge.

## Methods

Since the datasets except PDBBind and QM9 do not provide the 3D structure of molecules, we generate atom positions with the fast ETKDG method [51]. Although this method cannot give the best conformer for a molecule, it is very fast for conformer generation and conducive to large-scale molecule screening. We chose the atomic number, formal charge, chirality, number of bonded hydrogens atoms, hybridization, aromaticity, atomic mass, and hydrogen bond information as input atom features (detailed information can be seen in Additional file 1: Table S1), and all molecular manipulations were handled using RDKit and Pybel [52]. The 1-hop index, 2-hop index, and 3-hop index are generated by networkx [53]. After generating atom features, atom positions, and the indexes, the datasets are randomly split into train set, validation set, and test set with a split ratio of 8:1:1. The FFiNet model was implemented using PyTorch Geometric (PyG) [54] and PyTorch. In the training process, we optimize the model using Adam [55] with 128 molecules per mini-batch. We use early stopping [56] to avoid overfitting and reduce training time consumption. To give a fair comparison with the baselines, three independent runs with different random seeds are performed. Besides, we use the same input features for GATv2, GIN, GCN and the same training protocol for all GNNs. Since RF and SVM can only process tabular data, the Morgan fingerprint is generated to feed the models. The graph-based baselines are all implemented by DeepChem [57], and ML-based baselines were implemented by scikit-learn [58]. We use Bayesian optimization for hyperparameters search by using Hyperopt [59] package. The search space of FFiNet and baselines are shown in Additional file 1: Tables S2 and S3.

## Supplementary Information

**Additional file 1. **Additional model details, hyperparameters setting, and model interpretation.

## Data Availability

To ensure the reproducibility of the results, all data and the source code of all the models used in this work can be acquired at https://github.com/fate1997/FFiNet.
