# Supplementary material for: Force field-inspired molecular representation learning for property prediction
Source: J Cheminform. 2023 Feb 6;15:17. doi: 10.1186/s13321-023-00691-2 (PMC9901163; doi:10.1186/s13321-023-00691-2)
Supplement: Supplementary file 1 — Additional file 1. Additional model details, hyperparameters setting, and model interpretation. [file 13321_2023_691_MOESM1_ESM.docx]

**Supplementary Information for:**

**Force field-inspired molecular representation learning for property prediction**

Gao-Peng Ren^1,2^, Yi-Jian Yin^1,2^, Ke-Jun Wu*^1,2,3^, Yuchen He*^4^

^1^Zhejiang Provincial Key Laboratory of Advanced Chemical Engineering Manufacture Technology, College of Chemical and Biological Engineering, Zhejiang University, Hangzhou 310027, China

^2^Institute of Zhejiang University-Quzhou, Quzhou 324000, China

^3^School of Chemical and Process Engineering, University of Leeds, Leeds LS2 9JT, U.K. ^4^State Key Laboratory of Industrial Control Technology, College of Control Science and Engineering, Zhejiang University, Hangzhou, 310027, China

*Corresponding author: Ke-Jun Wu, K.Wu@zju.edu.cn; Yuchen He, hyc19940615@zju.edu.cn

# : Model details

## 1.1 Input features

Our model’s input atom features for datasets except for PDBBind are listed in Table 1.

Table 1 Input features of FFiNet^a^

| Feature | Description | Size |
| --- | --- | --- |
| atom type | Type of atoms (e.g. C, N, O), by atomic number | 38 |
| formal degree | number of covalent bonds | 6 |
| chirality tag | CW, CCW, unspecified or other | 3 |
| number of hydrogens | number of bonded hydrogen atoms | 6 |
| hybridization | Unspecified, sp, sp^2^, sp^3^, sp^3^d, sp^3^d^2^ or other | 8 |
| aromaticity | whether the atom is part of an aromatic system | 2 |
| hydrogen bonding | hydrogen bond donor and/or acceptor (binary values) | 2 |
| atomic mass | mass of the atom | 1 |

^a^All features are one-hot encodings except for atomic mass and hydrogen bonding

All these features are extracted by RDKit and DeepChem on the molecules without hydrogen. In order to save the time of conformer generation, we use ETKDG^1^ in RDKit to calculate atom positions. We have also tried another strategy to obtain atom positions, that is using MMFF94 to obtain the best conformers among 50 conformer candidates. The experiments show these two 3D geometry generation methods lead to similar results. Since our model considers the atoms within 3-hops with the target atom, except for the edge index (1-hop index), the indexes with lengths three (2-hop index) and four (3-hop index) also need to be generated. We utilize Networkx package^2^ and the recursive method to generate these indexes and call them 2-hop index and 3-hop index, respectively. The 1-hop index, 2-hop index, and 3-hop index are referred to the subgraph of length 1, 2, and 3 connectivity in COO format.

As for the atom features of PDBBind, since the generation of hydrogen bonding features is very time-consuming for large molecules, we remove them from the atom features of protein and ligand. To distinguish whether the atom is from protein or ligand, we add an indicator in the atom features that is zero for ligand and one for protein. We choose the pockets around the ligand as the protein graphs since the original protein graphs contain too many atoms and are difficult for preprocessing, besides, the atoms around the ligand affect the binding affinity most. The pockets often contain multiple molecules, which are amino acids and non-standard residues like water. We remove these non-standard residues by pdb-tools^3^. After cleaning the pocket and generating the atom features of the a protein and ligand, we concatenate the features and positions of the protein and ligand. Then the nonbonded edges and edge attributes are generated as introduced in the main body. Finally, large protein-ligand graphs just like the normal molecule graphs are generated and fed into the FFiNet.

## 1.2 Position encodings

One of the obstacles to expanding the receptive field is how to distinguish the atoms in different hops. We use two strategies to solve this problem, the first is injecting position encodings in the k-hop attention mechanism, second is the axial attention mechanism. The latter will be talked about in section 0. Position encoding is a method to inject some information about the relative or absolute position of the tokens in the sequence. In this work, we use sine and cosine functions of different frequencies as position encodings:

| $PE\left( pos,2i \right)=\sin\left( \frac{pos}{{10000}^{\frac{2i}{d^{'}}}} \right)$ | (1) |
| --- | --- |
| $PE\left( pos,2i+1 \right)=\cos\left( \frac{pos}{{10000}^{\frac{2i}{d^{'}}}} \right)$ | (2) |

where $pos$ is the position of a vector in a sequence, $d’$ is hidden channels, and $i$ is a number from zero to $d’$. We first treat the initial k-hop embeddings (k=0, 1, 2, 3) as a sequence. And multiplying position encodings on them. We do not use a sum operation like Transformer because the k-hop attention mechanism is not sensitive to the sum operation.

## 1.3 k-hop output

After calculating the attention scores by Eq. (2) in the main text, the softmax function is applied for normalization:

| $\alpha_{ij}^{k}=\mathrm{softmax}\left( e_{ij}^{k} \right)=\frac{\exp\left( e_{ij}^{k} \right)}{\sum_{j^{'}\in\mathcal{N}_{k}\left( i \right)} \exp\left( e_{ij^{'}}^{k} \right)}$ | (3) |
| --- | --- |

where $i$ is the target node, $j$ is one of the k-hop neighbors of node $i$, $\mathcal{N}_{k}(i)$ denotes the k-hop neighbors of node $i$,$e_{ij}^{k}$ denotes the k-hop attention score, $\alpha_{ij}^{k}$ denotes the normalized attention scores. Then the node embeddings are updated by a weighted average of the transformed features of the k-hop neighbors using the normalized attention coefficients:

| $\boldsymbol{h}_{i}^{k}=\sum_{j\in\mathcal{N}_{k}\left( i \right)} \alpha_{ij}^{k}\boldsymbol{W}_{\boldsymbol{k}}\boldsymbol{h}_{j}$ | (4) |
| --- | --- |

Where $h_{i}^{k}$ denotes the k-hop output for atom $i$, and $\boldsymbol{W}_{\boldsymbol{k}}\boldsymbol{h}_{j}$ denotes the projected node embeddings.

## 1.4 Axial attention

After calculating the k-hop outputs (k is 1, 2, 3), the next step is to merge these vectors into one vector. There are two simple ideas that we can concatenate or sum these outputs. Both ideas are tested in different tasks, however, they can neither perform well. The reason why the concatenation operation doesn’t work is probably that the concatenation will break the consistency between input vectors and their hidden states and will cause the dimension of the hidden state to grow exponentially. As for the summation operation, since the attention score of different hops is normalized separately, it will make it impossible to distinguish the outputs from different hops. Another way to aggregate these outputs is to treat the initial embeddings and the outputs as a sequence and generate the final output according to the importance of each hop output to the target atom. We use the self-attention mechanism which is similar to Transformer to calculate the attention score, and we called this attention mechanism axial attention. To decrease the number of parameters to be learned, we apply linear projection only on k-hop outputs. The axial attention score is:

| $e_{\mathrm{axial}}^{k}\left( h_{i}^{k}, h_{i} \right)=\frac{{\boldsymbol{h}_{i}(\boldsymbol{W}}_{\mathrm{axial}}^{k}\boldsymbol{h}_{i}^{k})^{T}}{\sqrt{d^{'}}}$ | (5) |
| --- | --- |

where $e_{\mathrm{axial}}^{k}$ denotes the axial attention score of the target atom $i$ and its k-hop output $\boldsymbol{h}_{i}^{k}$, $\boldsymbol{h}_{i}$ is the initial embeddings of the target node $i$, $\boldsymbol{W}_{\mathrm{axial}}^{k}$ denotes the learned parameters for the k-hop output. Then we apply the softmax function to get a normalized axial attention score, $\alpha_{\mathrm{axial}}^{k}$. Then the output of the axial attention module is:

| $\boldsymbol{H}=\sum_{k=1,2,3} \alpha_{axial}^{k}\boldsymbol{H}^{k}$ | (6) |
| --- | --- |

where $\boldsymbol{H}^{k}\mathbb{\in R}^{n\times d^{'}}$ denotes the stack of $h_{i}^{k}$, $n$ is the number of atoms in a molecule. Since the k-hop attention includes multi-head attention, we concatenate these outputs of different heads for the layers except for the last layer. Then apply the activation function to the output.

## 1.5 Dropout, residual, and layer normalization

To prevent overfitting, we apply dropout in the FFiNet. It is worth noting that there are four types of attention scores in our work. Therefore, there are four dropout modules in the model. We experiment with using different values or the same values to the dropout modules and use the Bayesian optimization method to optimize the hyperparameters. The results show that the two methods get similar performance on different tasks, therefore, to reduce the model hyperparameters, we use the same values on all the dropout modules in the model.

The biggest difficulty in limiting the number of layers of GNNs is the over-smoothing problem. There have been many methods to address this problem, and among them, the residual connection is the most commonly used method. The residual connection is originally proposed in the ResNet, and proved to be very useful for the deeper neural network. And the residual connection has been proved useful to alleviate the over-smoothing problem^4^. Therefore, in this work, we adopt the residual connection that adds the initial embeddings of nodes to the output node embeddings.

Since the batch size of the graph neural network for prediction is usually small, we did not use batch normalization to accelerate the convergence rate of the model. The layer normalization is a substitute for batch normalization, which normalizes the examples along the layer dimension. In this work, we use layer normalization before applying the activation function to the output.

# : Hyperparameters optimization

## 2.1 FFiNet

For each task, we apply Bayesian optimization by the hyperopt package. The search space of hyperparameters is listed in Table 2.

Table 2 Hyperparameters optimized for FFiNet on each task

| Hyperparameters | Search space |
| --- | --- |
| Hidden dimension | 16, 32, 64, 96 |
| Hidden layers | 1, 2, 3, 4, 5 |
| Number of heads | 4, 8, 12 |
| Activation | PReLU, ELU |
| Dropout | 0.1, 0.2, 0.3 |
| Prediction layers | 1, 2 |
| Prediction dropout | 0.1, 0.2 |
| Prediction hidden dimension | 256, 512 |
| Batch size | 128 |
| Learning rate | 5e-4, 2e-3 |

## 2.2 Baselines

For the GATv2, we use the same search space with FFiNet, since they are all attention-based approaches and have the same types of hyperparameters. For the GCN, GIN models, we use the same prediction module as FFiNet since they are originally for node classification tasks and have no readout phase and prediction module. For the DimeNet, we use the default setup for the hyperparameters for spatial embedding. Finally, the search space of GCN, GIN, DimeNet, and SVM are listed in Table 3. Besides, for the RF model, we set the estimator 500.

Table 3 Hyperparameters optimized for baselines on each task

| Model | Hyperparameters | Search space |
| --- | --- | --- |
| GIN | Number of layers | 1, 2, 3 |
|  | Hidden dimension | 32, 64, 128 |
| GCN | Number of layers | 1, 2, 3 |
|  | Hidden dimension | 32, 64, 128 |
| DimeNet | Number of blocks | 1, 2, 3 |
|  | Hidden dimension | 32, 64, 128 |
| SVM | Regularization parameter | 0.25-4 with step 0.25 |
|  | Kernel coefficient | 0.02-0.2 with step 0.02 |

# : Dive into FFiNet

## 3.1 Molecule length

The characteristic of FFiNet architecture heralds FFiNet may be good at large molecule modeling. To confirm this conjecture, we studied the relationship between the model performance and molecule size. We treat the longest path of a molecule as molecule length, taking Lipophilicity and BBBP as examples, the molecule lengths of most molecules in datasets are greater than 10 (as shown in Figure 1a and Figure 1c). Since our model is derived from GATv2, we choose it as a baseline model to investigate the relationship between performance and molecule length. Figure 1b and Figure 1d show that the performance improvement of FFiNet over GATv2 is more significant on longer molecules in general. This result may be explained by two reasons: First, FFiNet with the same number of layers as GATv2 can propagate longer information; second, there are more nonbonded interactions when the molecule is longer and the FFiNet can handle the message passing between nonbonded atoms more efficiently. The results show that FFiNet may be more suitable for the prediction of the property of longer molecules.


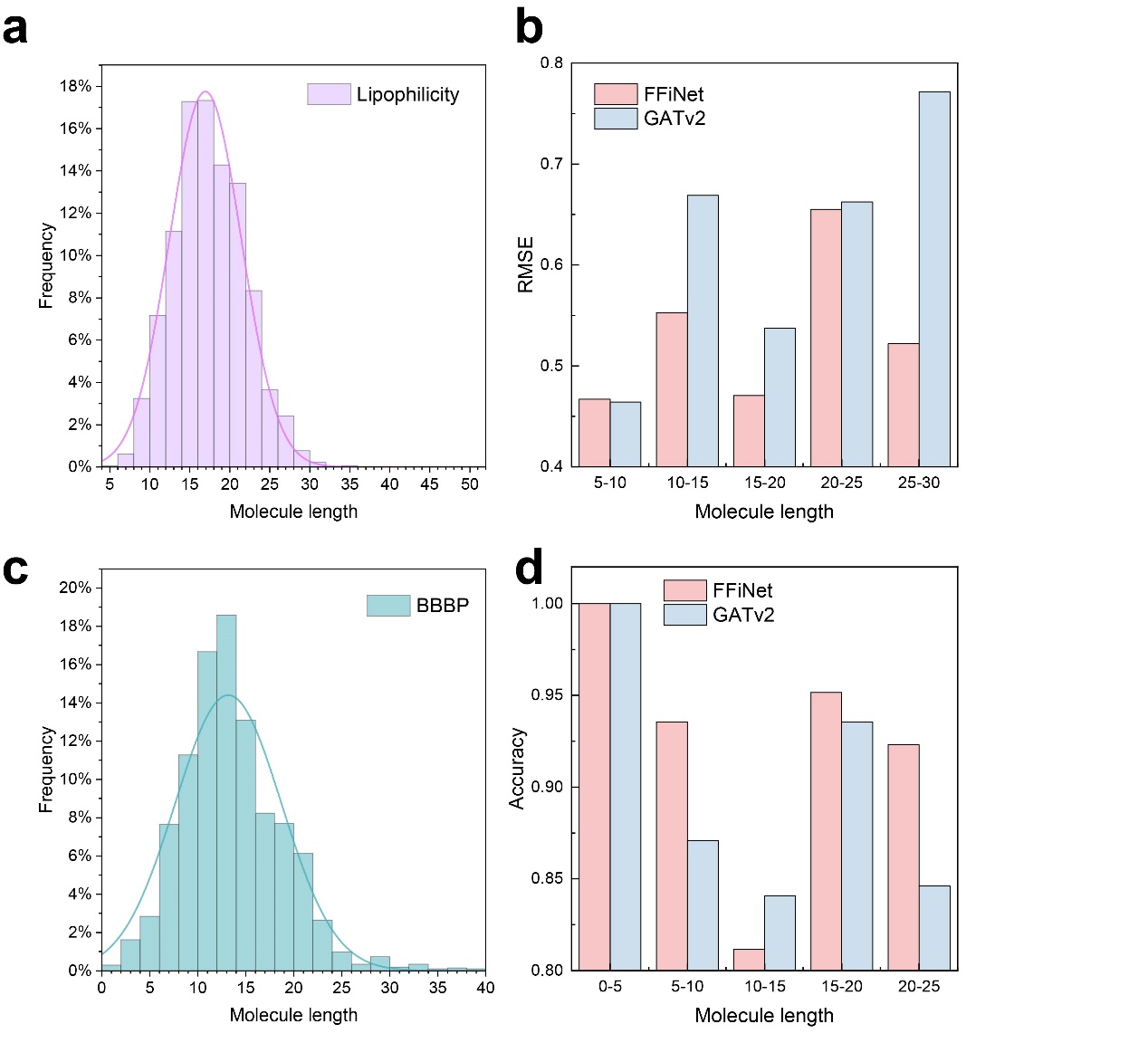


Figure 1 Frequency and performance at different molecule length (longest path of a molecule) on Lipophilicity and BBBP datasets. **a**, Frequency on Lipophilicity dataset. **b**, Performance on Lipophilicity dataset (lower is better). **c**, Frequency on BBBP dataset. **d**, Performance on BBBP dataset (higher is better).

## 3.2 Spatial embedding memory

We check the number of parameters and FLOPs of spatial embedding modules in FFiNet and DimeNet (Table 4). We use the same hyperparameters (such as the number of RBF basis) of the spatial embedding module in DimeNet. It can be shown that the number of parameters and FLOPs of the spatial embedding module in FFiNet is much lower than that in DimeNet.

Table 4 The number of parameters and FLOPs of DimeNet and FFiNet

|  | Spatial embedding module | |
| --- | --- | --- |
| Model | Number of parameters | FLOPs |
| DimeNet | 6912 | 668160 |
| FFiNet | 2688 | 148864 |

## 3.3 Receptive field

In this work, the receptive field is set to three hops. The former discussion in the main text has shown the perceptual field less than 3-hop cannot get better performance than 3-hop. To further prove it is reasonable to choose three hops as the receptive field, we plot the atom-pair distances of different hops of the molecules in the lipophilicity dataset (Figure 2). As shown, the distance of most 1-hop atom pairs (bonded atom) is less than 1 Å, the distance of most 2-hop atom pairs is around 2.5 Å, and the range of 3-hop atom pair distance is very large (from 2 Å to 4.25 Å). The nonbonded interactions are very small when the atom distance is longer than 5.0 Å^5^, which is also consistent with the cutoff distance in the SchNet, DimeNet, SphereNet, and so on. Therefore, it is reasonable to set the perceptual field as three hops.


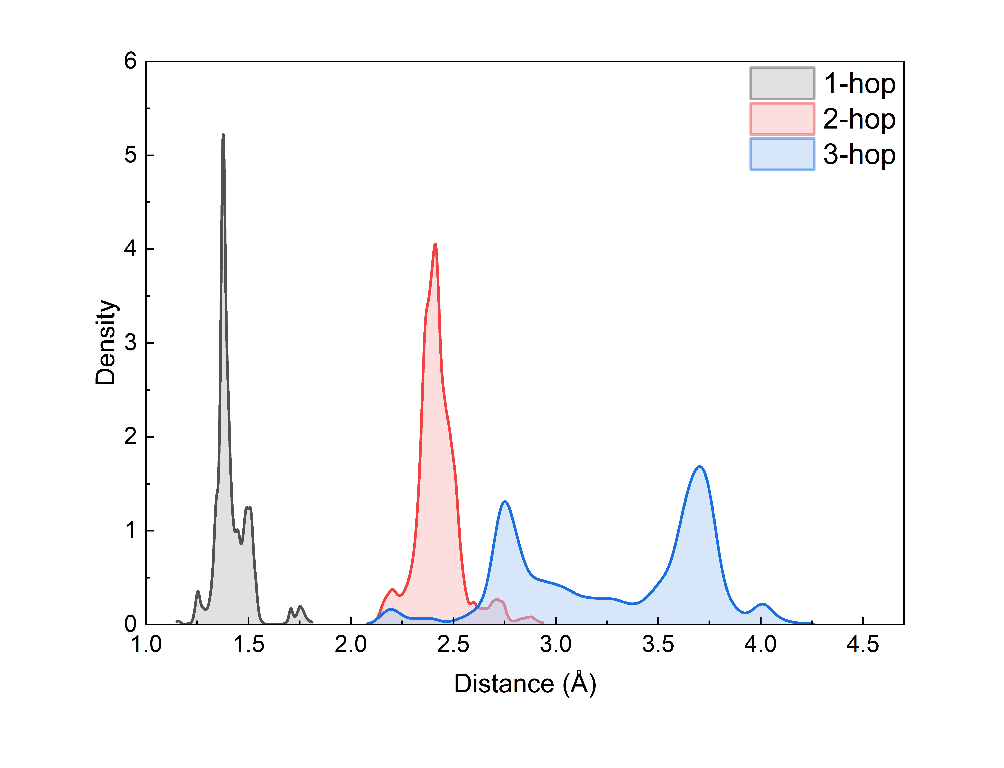


Figure 2 The distribution of atom pair distance of 1-hop to 3-hops in the molecules of the lipophilicity dataset

# : Additional visualization

## 4.1 Atom similarity

To give more evidence that the FFiNet can distinguish between different functional groups for given property, we plot more heat maps of the atom similarity matrix (Figure 3, Figure 4, Figure 5) on the Lipophilicity dataset. As Figure 3 shows, the similarity between the atoms on the carbon chain and those on the benzene ring is relatively small, which shows the FFiNet can distinguish rings and chains in the molecule. And the oxygen atom does not have a high similarity with other atoms in the molecule, which shows the FFiNet can distinguish different types of atoms. More evidence can be found in Figure 4. The nitrogen atom and the oxygen atom with an index of two show low similarity with other atoms (most are carbon atoms), and the similarity between the oxygen atom with an index of nine and the carbon atoms in the benzene rings is relatively low than carbon atom pairs. Moreover, as Figure 5 shows, there are two parts with high similarity and they correspond to the five-element ring and six-element ring, respectively, which shows the FFiNet can distinguish the different rings.


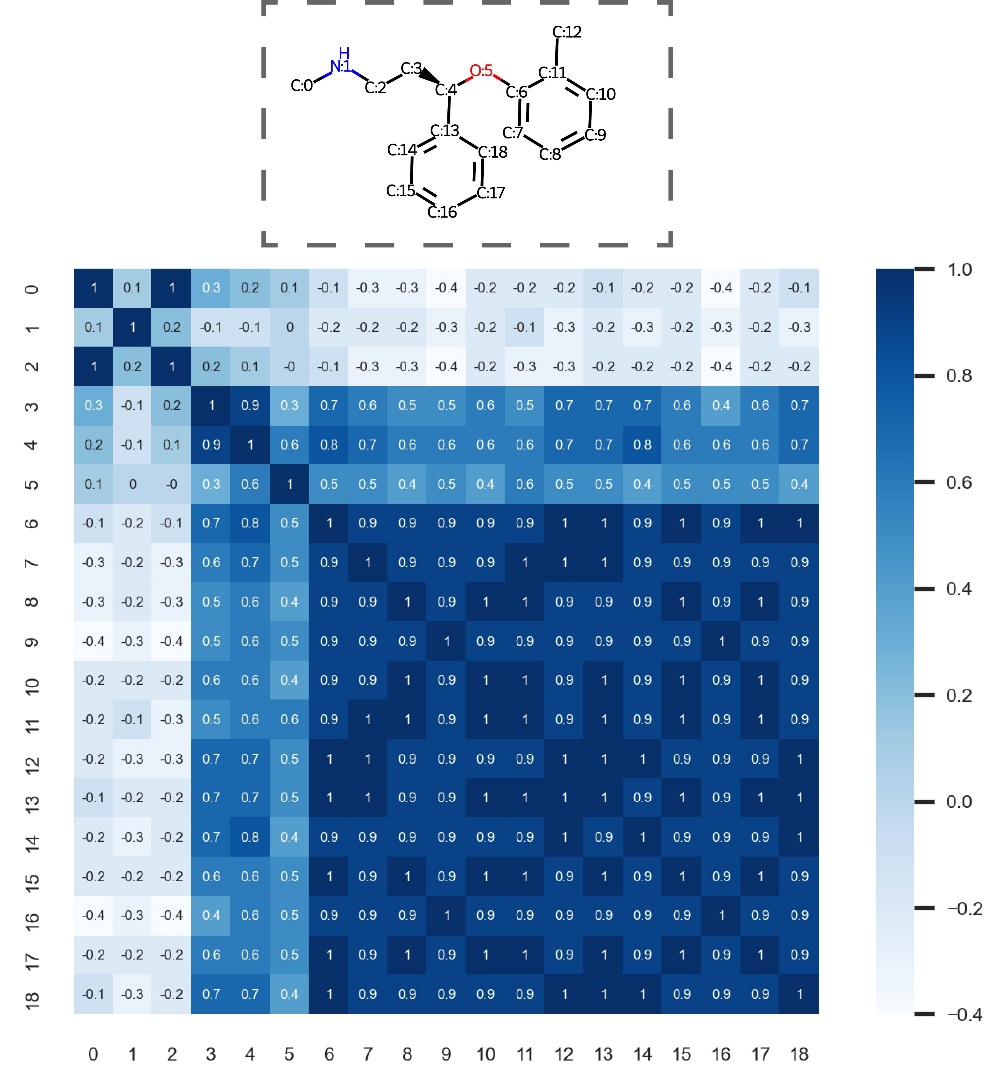


Figure 3 Heat map of the atom similarity matrix for Atomoxetine


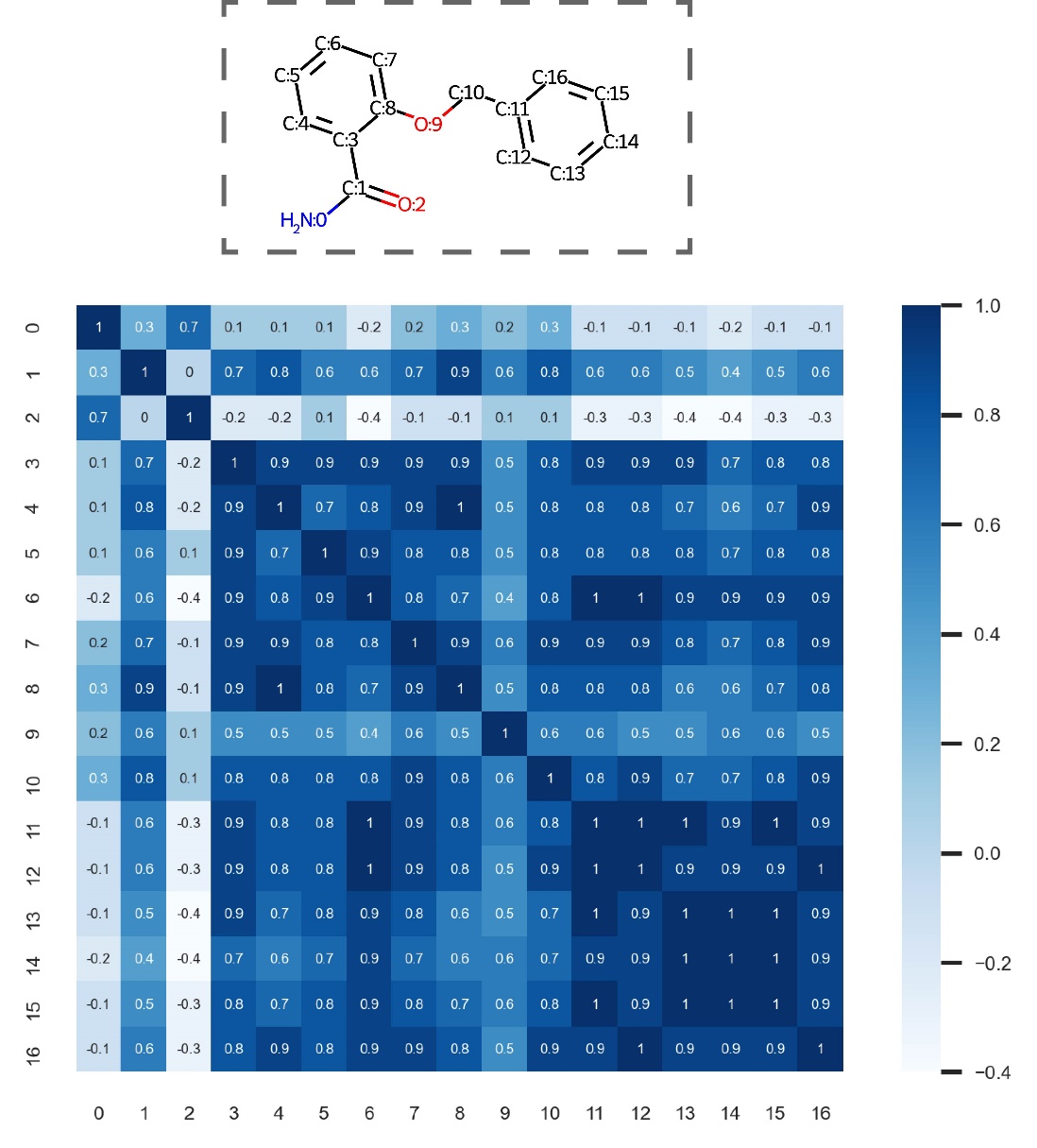


Figure 4 Heat map of the atom similarity matrix for 2-(phenylmethoxy)benzamide


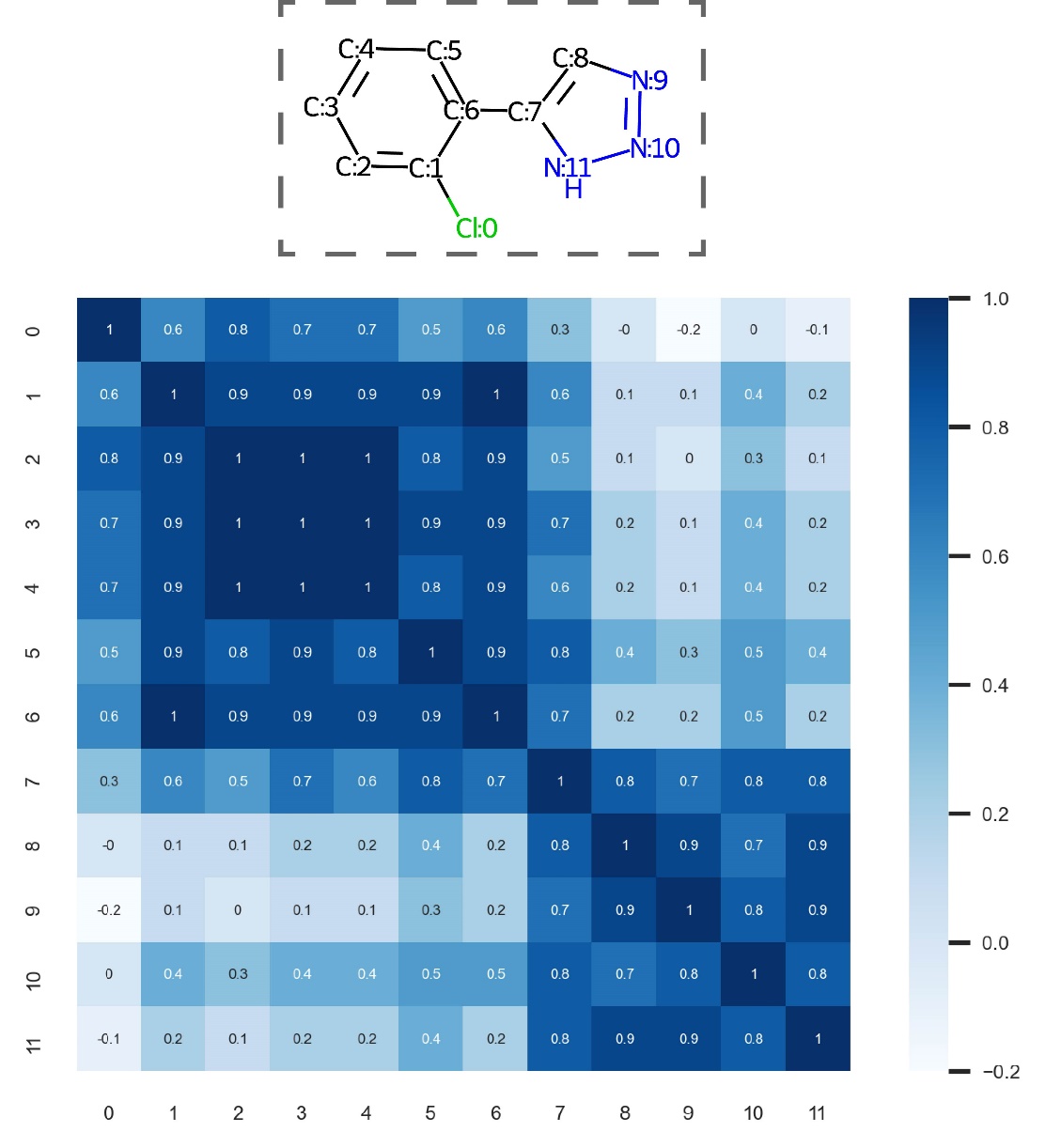


Figure 5 Heat map of the atom similarity matrix for the molecule with smiles of ‘Clc1ccccc1-c1cnn[nH]1’

## 4.2 Attention weights and atom contributions

We also plot additional heat maps of atom contribution (Figure 6). As shown, the first molecule assigns big values for sulfur and nitrogen atoms and the second molecule assigns big values for oxygen and nitrogen atoms. These atoms involve the typical hydrophilic group and lipophilic group, which indicates that the FFiNet can correctly find the important atoms for the specific molecule property.


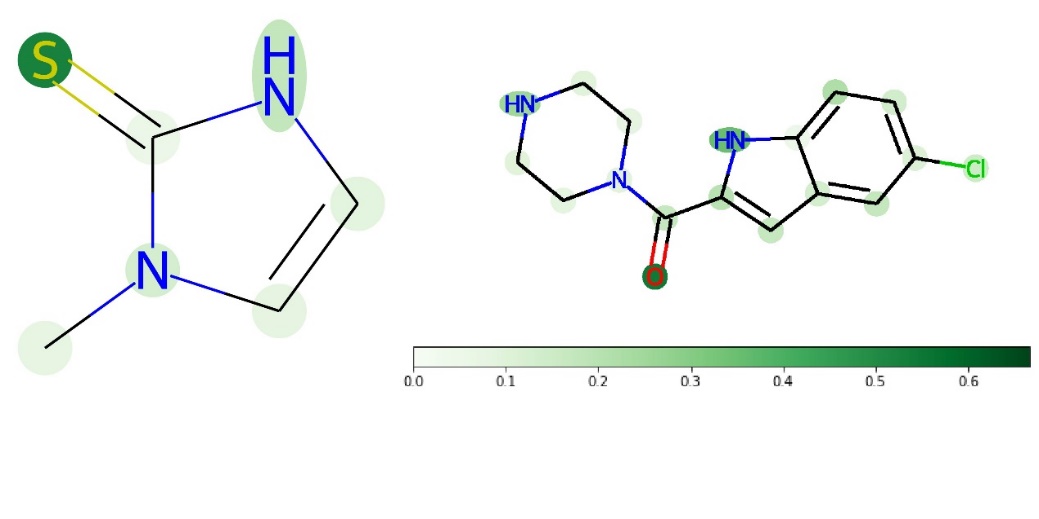


Figure 6 The atomic contributions for the lipophilicity.

# References

1. Riniker, S. & Landrum, G. A. Better Informed Distance Geometry: Using What We Know To Improve Conformation Generation. *J. Chem. Inf. Model.* **55**, 2562–2574 (2015).

2. Hagberg, A. A., Schult, D. A. & Swart, P. J. Exploring network structure, dynamics, and function using NetworkX. *7th Python Sci. Conf. (SciPy 2008)* 11–15 (2008).

3. Rodrigues, J. P. G. L. M., Teixeira, J. M. C., Trellet, M. & Bonvin, A. M. J. J. pdb-tools: a swiss army knife for molecular structures. *F1000Research* **7**, 1961 (2018).

4. Li, G., Xiong, C., Thabet, A. & Ghanem, B. Deepergcn: All you need to train deeper gcns. *arXiv Prepr. arXiv2006.07739* (2020).

5. Kitaigorodsky, A. I. Non-bonded interactions of atoms in organic crystals and molecules. *Chem. Soc. Rev.* **7**, 133–163 (1978).
